# Effectiveness on Frailty of an eHealth-Based Rehabilitation Program in Older People with Acute Heart Failure and/or Acute Coronary Syndrome: Study Protocol for a Randomized Trial and Baseline Data of Participants

**DOI:** 10.3390/jcm15072573

**Published:** 2026-03-27

**Authors:** Gaia Cattadori, Roberto F. E. Pedretti, Simona Sarzi Braga, Gabriele Maria Maglio, Monica Mancino, Tiziana Staine, Sara Mondaini, Luana Eramo, Valeria Pellegrini, Rosalba La Grotta, Denise Bruno, Eros Patuzzo, Giulia Matacchione, Angelica Giuliani, Rosa Carbonara, Angela Ferrulli, Maria Venneri, Chiara Osella, Lucrezia Quarto, Maddalena Genco, Irene D’Addabbo, Francesca Camicia, Lucia Palazzo, Attilio Caruso, Liana Spazzafumo, Fabiola Olivieri, Elena Tagliabue, Francesco Prattichizzo, Andrea Passantino

**Affiliations:** 1 IRCCS MultiMedica, 20138 Milan, Italy; gaia.cattadori@multimedica.it (G.C.);; 2 Department of Clinical Sciences and Community Health, Section of Cardiology, University of Milan, 20122 Milan, Italy; 3 Clinic of Laboratory and Precision Medicine, IRCCS INRCA, 60121 Ancona, Italy; 4 Department of Clinical and Molecular Sciences, Universita Politecnica Delle Marche, 60100 Ancona, Italy; f.olivieri@inrca.it; 5 Cardiac Rehabilitation Unit of Bari Institute, Istituti Clinici Scientifici Maugeri IRCCS, 70124 Bari, Italyandrea.passantino@icsmaugeri.it (A.P.); 6 Bioengineering Unit of Bari Institute, Istituti Clinici Scientifici Maugeri IRCCS, 70124 Bari, Italy; 7 Scientific Direction, IRCCS INRCA, 60121 Ancona, Italy; 8 Biogerontology Center and Geriatric Mouse Clinic, IRCCS INRCA, 60121 Ancona, Italy

**Keywords:** cardiovascular diseases, frailty, telemedicine, cardiac rehabilitation, protocol

## Abstract

**Background**: Frailty is highly prevalent among older adults with cardiovascular disease (CVD) and strongly predicts disability and mortality after cardiac events. Although cardiac rehabilitation (CR) improves prognosis, frail older patients often face barriers to participating in in-person programs. eHealth-based, home-delivered CR programs incorporating tele-rehabilitation and remote monitoring may improve accessibility, yet evidence regarding their effectiveness on frailty status remains limited. **Methods**: We designed a multicenter, randomized, parallel-group trial enrolling people ≥65 years recently hospitalized for acute heart failure (AHF) and/or acute coronary syndrome (ACS). Participants were randomized 1:1 to an eHealth home-based tele-rehabilitation program or the usual care. The primary endpoint is frailty prevalence at follow-up, defined by an Essential Frailty Toolset (EFT) score ≥3, with co-primary outcomes being between-group differences in the mean levels of EFT and Short Physical Performance Battery (SPPB) scores after 3–6 months. Secondary endpoints include mortality and hospitalization, among others. **Results**: The full protocol and study procedures are reported. Between May 2024 and December 2025, 589 patients were screened at the two Italian centers involved; 442 met eligibility criteria and 209 were enrolled and randomized. Baseline characteristics were largely comparable between groups. The mean age was 77 ± 9 years, 70% were male, and 55% had ACS. Lower-than-expected enrollment was mainly attributable to refusal related to difficulties in using digital devices. **Conclusions**: This randomized trial will evaluate whether a multidomain, eHealth-based CR intervention can reduce the prevalence or degree of frailty in older people after AHF or ACS. We report the study protocol and baseline characteristics of the enrolled cohort, highlighting the challenge of digital illiteracy in contemporary older populations.

## 1. Introduction

Older people with cardiovascular disease (CVD) are particularly vulnerable, exhibiting both high mortality [1] and a consistent risk of disability following hospitalization [2]. A major determinant of such poor outcomes is frailty, a clinical syndrome highly prevalent among older individuals with CVD [3]. Frailty is characterized by a progressive decline in the physiological reserves of multiple organ systems, which limits the individual’s ability to cope with stressors and predisposes them to negative health trajectories. As an independent predictor of autonomy loss, frailty is strongly associated with adverse clinical outcomes, including hospital readmissions, institutionalization, and death [4].

Preliminary evidence suggests that frailty may not represent an irreversible condition, raising the possibility that targeted interventions could improve resilience and potentially alter the course of the syndrome [3]. Cardiac rehabilitation (CR) programs are established as effective tools to improve cardiovascular prognosis and quality of life [5]. However, despite their proven efficacy, the uptake of CR remains suboptimal, with older and frail patients facing the greatest barriers due to mobility limitations, comorbidities, and thus a limited access to structured programs. In this respect, digital health interventions offer the opportunity to extend the reach and adaptability of traditional models. By facilitating adherence, enhancing accessibility, and supporting individualized care, eHealth-based CR programs, which incorporate tele-rehabilitation (TR) and remote monitoring, have the potential to overcome many of the barriers encountered by older patients [6]. However, few studies have explored the efficacy of eHealth home-based CR interventions on frailty. Indeed, a recent systematic review concluded that more studies are needed to define its efficacy and usefulness to make definitive recommendations [7].

To fill this gap, we undertook a randomized trial to evaluate whether an eHealth home-based CR intervention, compared with the standard of care, is able to reduce the rate or the degree of frailty in people ≥ 65 years and with acute heart failure (AHF) and/or acute coronary syndrome (ACS). Here we report the protocol of the trial and the baseline clinical data of included participants.

## 2. Materials and Methods

### 2.1. Study Design

**Figure 1:**
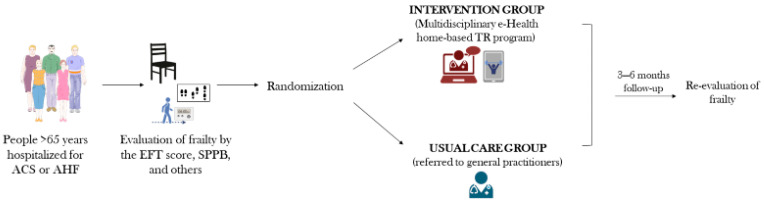
**Schematic summary of the design of the trial.** This multicenter, randomized, parallel-group study enrolled older patients recently hospitalized for acute heart failure (AHF) and/or acute coronary syndrome (ACS) at two Italian sites: IRCCS MultiMedica in Sesto San Giovanni (Milan) and the Istituti Clinici Scientifici Maugeri IRCCS, Cardiac Rehabilitation Unit of the Bari Institute in Bari. Participants aged ≥65 years with AHF or ACS were screened for frailty with multiple tools including Essential Frailty Toolset (EFT) and the Short Physical Performance Battery (SPPB) and then randomly assigned to one of two groups: Group A, receiving an eHealth-supported home-based tele-rehabilitation (TR) program, or Group B, receiving usual care. After a minimum of 3 and up to a maximum of 6 months, all enrolled subjects underwent a follow-up assessment of frailty.

This is a multicenter, randomized, parallel-group study in older patients with a recent hospitalization for AHF and/or ACS, conducted at two study sites: IRCCS MultiMedica, Sesto San Giovanni (MI), Italy, and Istituti Clinici Scientifici Maugeri IRCCS, Cardiac Rehabilitation Unit of Bari Institute, Bari, Italy. People with AHF or ACS and ≥ 65 years old were randomized into two groups: Group (A), a multidisciplinary eHealth home-based TR program; and Group (B), which received the usual care. After a minimum of 3 and up to a maximum of 6 months, enrolled subjects underwent a new evaluation for frailty. The duration of follow-up was flexible to facilitate participation and adherence. The latest available follow-up will be considered for each individual and mixed models will be used to account for different times to follow-up in the analyses. A summary of study design is presented in Figure 1. Given the nature of the study involving an active intervention only in the group assigned the eHealth arm, the trial was not blinded, i.e., investigators performing the frailty screening both at baseline and follow-up were also involved in the development of the program. The help of a caregiver was allowed but was not mandatory to participate to the study. Caregivers were not in charge of exercise supervision.

### 2.2. Study Endpoints

#### 2.2.1. Primary Endpoints

The primary endpoint is to assess whether a home-based TR program significantly reduces the prevalence of frailty in comparison to usual care in older individuals hospitalized for ACS and/or AHF. Frailty status is defined as having an Essential Frailty Toolset (EFT) score (ranging from 0 to 5) ≥ 3 [8].

Since the recent literature suggests that frailty is a progressive continuum and not a present/absent condition [9,10] and considering that small improvements in the degree of frailty are associated with tangible benefits [11], the mean degree of frailty was considered as a co-primary endpoint. In particular, mean differences between groups at the end of treatment in the EFT and Short Physical Performance Battery (SPPB) scales were set as co-primary outcomes after the start of the enrollment due to the considerations reported above. After termination of the enrollment, we observed that the study is powered to detect a difference in these co-primary outcomes, while the power for the primary outcome of frailty prevalence is lower than initially planned. Thus, this and the other secondary endpoints will be considered as exploratory.

#### 2.2.2. Secondary Endpoints

- A composite endpoint of all-cause death and all-cause hospitalizations.
- A composite endpoint of cardiovascular death and cardiovascular hospitalizations (ACS, AHF, cardiac arrhythmias, cerebrovascular events, peripheral arterial vascular event).
- Changes in walking distance during 6 min walk test at follow-up vs. baseline in the two groups.
- Assessment of the quality of life, comorbidity burden, nutritional and cognitive status, depression, adherence to medical therapy and anthropometric measures. Tools for the evaluation of these endpoints are described below.
- Incidence of falls during follow-up.
- Proteomic and miRNOmic analyses to search for biomarkers of frailty.

### 2.3. End of Study Definition

The end of the study is defined as the date of the last visit for the last subject in the study. A participant is considered to have completed the study if they have completed all steps of the protocol.

### 2.4. Study Population

#### 2.4.1. Inclusion Criteria

- Age ≥ 65 yrs. The protocol was modified after the start of the study to lower the previous threshold of 75 years due to the high rate of refusals to participate attributable to the self-reported inability of older individuals to use technological devices (as detailed below).
- Recent (<30 days) hospitalization for AHF or ACS.
- Signed informed consent.

#### 2.4.2. Exclusion Criteria

- Judgment by the investigator that the participant is unlikely to comply with study procedures (i.e., ability of patient or caregiver in utilizing eHealth device).
- Other medical conditions determining a ≤6-month survival prognosis.
- Severe cognitive impairment, assessed through Mini Mental State Examination (MMSE < 15).
- Participation in another clinical study with a study intervention administered in the last 4 weeks.

### 2.5. Study Procedures

Step 1. Patients were screened and recruited during hospitalization in the Cardiovascular Department of IRCCS MultiMedica (Sesto San Giovanni—MI, Italy) or Cardiac Rehabilitation Unit of IRCCS ICS Maugeri (Bari—BA, Italy). The main clinical and demographic data were collected for each patient. The day before discharge, patients performed a 6 min walk test, gave a blood sample to test for molecular biomarkers and underwent a frailty evaluation on both the EFT and SPPB scales. Depression, quality of life, cognitive status, nutritional status, and adherence to medical therapy were evaluated with adequate tools. Blood samples were collected to identify biomarkers able to identify frailty and predict its progression.

Step 2. Before discharge, all enrolled patients were randomized in a 1:1 ratio to intervention (Group A) or usual care (Group B). Group A followed a multidisciplinary home-based TR program while Group B was referred to general practitioners (GPs).

Step 3. The home-based TR program was implemented in Group A patients. Group B was referred to GPs according to usual care. During step 3, clinical outcomes and adverse events were collected.

Step 4. After at least 3 and at most 6 months of follow-up, patients underwent final evaluation; the procedures of step 1 were repeated. Study procedures are summarized in Supplementary Table S1.

### 2.6. Description of Procedures

#### Measure of Frailty: EFT and SPPB

The EFT is a brief four-item (chair rise, cognitive status, hemoglobin, and serum albumin) frailty scale [8]. The EFT is scored 0 (least frail) to 5 (most frail). The prevalence of frailty was defined according to the EFT score, specifically EFT ≥ 3 out of 5.

The SPPB is an objective tool measuring the physical performance status with recognized prognostic values for multiple frailty-related outcomes [12,13,14]. The SPPB is calculated by the time spent or needed to complete multiple tasks: standing balance, usual gait speed, and five chair tests. The timed results of each subtest are rescaled according to predefined cutoff points for obtaining a score ranging from 0 (most physically frail) to 12 (least frail/best performance) [14,15].

### 2.7. Human Biological Sample Biomarker Collection and Analysis

The collection of samples for biomarker research is part of this study. Plasma was collected from all participants in this study at baseline and was collected at the end of follow-up.

To identify novel biomarkers able to identify frailty and predict its progression, we used plasma samples collected during the frailty evaluation to identify miRNAs and inflammatory proteins cross-sectionally associated with frailty. Such candidates are derived from an unbiased miRNOmic analysis and a focused proteomic analysis. Statistical analysis revealed which markers (and relative combinations) identify a frail status. Then, after dosing such candidate molecules in the whole cohort, we performed statistical analyses to explore if such markers are also able to predict the eventual improvement or deterioration in frailty at follow-up, after intervention.

### 2.8. Six-Minute Walk Test (6MWT)

The 6MWT was performed in an indoor 60 m long corridor, according to the recommendations of the American Thoracic Society [16]. All patients were instructed to walk along the corridor from one end to the other at their own pace, as many times as possible in the permitted time. After 6 min had elapsed, the patients were instructed to stop walking, and the total distance walked was determined. This test was supervised by a physical therapist who encouraged the patients in a standardized fashion at regular intervals.

### 2.9. Measure of Quality of Life: EuroQol (EQ) Visual Analog Scale (VAS)

The EQ VAS is a measure of self-reported health outcomes, based on a visual analog graduated scale (0 to 100) for assessing health status, ranging from the worst imaginable health state (0) to the best imaginable health state (100) [17].

### 2.10. Measure of Comorbidity: Cumulative Illness Rating Scale (CIRS)

Pre-existing comorbidities were assessed using the CIRS, which gives a severity score and comorbidity score. This index is based on the scoring (from 0 to 4) of disease severity for 14 items corresponding to organs that may be affected by chronic disease. The CIRS severity score can be calculated as the average of each CIRS item score. The CIRS comorbidity score is based on the count of organ systems with moderate-to-severe impairment [18].

### 2.11. Measure of Nutritional Status: Mini Nutritional Assessment (MNA)

The MNA is a screening tool aimed at assessing nutritional status in older patients through 18 questions in 4 areas (basic anthropometrics, dietary intake, global indicators and self-assessed health status). Individuals with a score of 24–30 are considered to have a normal nutritional status, a score of 17–23.5 suggests a risk of malnutrition, and a score of <17 identifies malnutrition [19].

### 2.12. Cognitive Status: Mini Mental State Examination (MMSE)

Global cognitive function was assessed by the MMSE. In clinical practice, MMSE is used to detect cognitive impairment, monitor cognitive decline over time, and evaluate the impact of potential treatments on cognition (particularly in older adults). It is brief, easily administered, and quickly scored. The measure assesses orientation, attention and calculation (Serial 7s, spell “world” backward), language (naming, repetition, comprehension, reading, writing, copying), and immediate and delayed recall. Scores > 24 indicate normal cognitive status, while lower scores indicate cognitive impairment [20].

### 2.13. Depression: Geriatric Depression Scale (GDS)

The GDS is a self-administered test developed for a brief screening of depression in older persons. The 15-item short form (GDS-15) was used for this study [21]. Scores may range from 0 (no depression) to 15 (severe depression). Answers are dichotomic to facilitate understanding and answering in older individuals. The questions can be read to patients. The GDS correlates highly with well-studied measures such as the Beck Depression Inventory, Zung Depression Inventory, and Hamilton Rating Scale for Depression [21].

### 2.14. Functional Independence: Barthel Index for Activities of Daily Living (ADLs)

The Barthel Index measures functional disability in 10 ADLs by quantifying patient performance. Five-point increments are used in scoring, with a maximal score of 100 indicating full independence whilst the lowest score of 0 indicates a patient with a completely bed-bound state [17].

### 2.15. Adherence to Therapy: Morisky Medication Adherence Scale (MMAS-8)

MMAS-8 is an 8-item, structured, self-measured, self-reported measure that assesses medication adherence [22].

### 2.16. Anthropometric Measures

The patients were weighed before breakfast without their shoes, and Body Mass Index (BMI) was calculated as [weight (kg)]/[height (m)]^2^.

### 2.17. Laboratory

Complete blood counts and creatinine, total cholesterol, hemoglobin, albumin, and NT-proBNP levels were measured as in routine clinical practice. The Glomerular Filtration Rate (eGFR) was estimated by the Cockcroft–Gault formula.

### 2.18. eHealth Home-Based CR Program

A multidisciplinary home-based CR program was implemented for Group A patients. The program lasted a maximum of 6 months; it was coordinated by a cardiologist and it was based on a multidisciplinary intervention by different healthcare providers: a nurse (case manager), physiotherapist, psychologist, and dietician.

The protocol was shared between the investigators involved in the two centers and was developed based on the most recent guidelines [5]. The online platform used for the intervention had a pre-specified list of questions and items to check, including the vital parameters to be collected at each visit, in order to maximize standardization and the fidelity of the programs between the two centers involved.

In the present study a synchronous model of cardiac TR, based on a real-time interaction between the patient and healthcare provider, was used. The advantages of this approach are that it allows a very close follow-up and better program personalization.

The core of the TR program was represented by scheduled video-calls with the patient (2 per week, 30–45 min duration each) by the nurse and physiotherapist focused on: education and cognitive training, exercise training session along with physiotherapy counseling once a week, assessment of healthy lifestyle, risk factor control, medication adherence and therapeutic targets. A video-call with a psychologist and dietician for psychosocial and nutritional counseling was provided at least once per fortnight. A social assistant was involved as needed by the nurse case manager.

The video-call was done by cell phone, tablet, or PC according to the patient’s preference.

For monitoring medication adherence and fall risk, one tool, i.e., text messaging, smartphone applications, or other electronic devices, tailored to the patient’s characteristics was applied. If applicable, an initial in-home visit to assess fall risk elements was also performed and a digital health alarm system to alert caregivers in case of physical discomfort was provided.

The program also included unscheduled calls from the patient in predefined time slots in case of need.

A pulse oximeter and a portable one-lead electrocardiograph for the telemonitoring of vital signs were available, and patients were able to call and be assisted in the case of urgent need or emergency, according to the internal standard operating procedure of each site.

Exercise training sessions were based on a ‘low level’ and a ‘high level’ of intensity, as detailed in the section below. The ‘low level’ consisted of 10–30 min of free exercise on a mini-ergometer (EverFit Welly M) without load and 30 min of callisthenic exercises, performed 3 times/week, and free walking twice a week. The ‘high level’ consisted of 30–45 min of free exercise on a mini-ergometer with incremental load, and 30–40 min of muscle reinforcement exercises using weights between 0.5 and 2.5 kg.

### 2.19. Exercise Training Protocol

**Figure 2:**
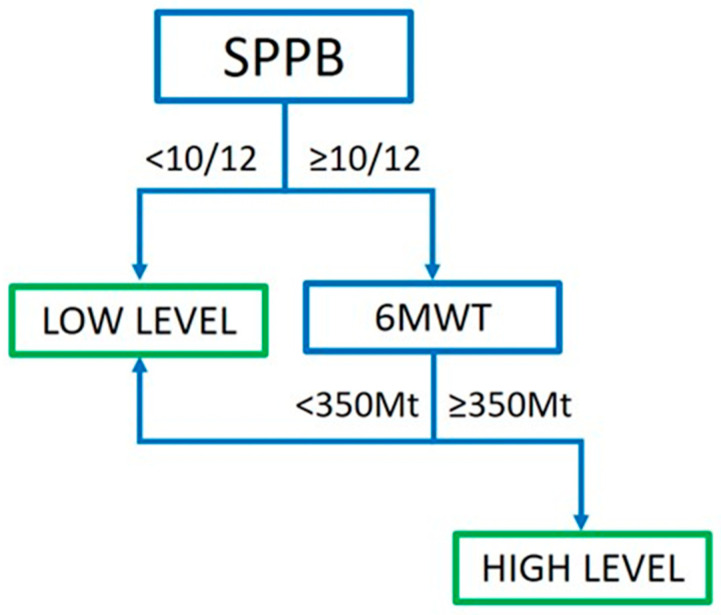
**Decision tree to assess the ability of subjects to complete a low- or high-intensity program of physical therapy.** People were tested with Short Physical Performance Battery (SPPB). In the case of an SPPB score < 10, people were prescribed a low-level physiotherapy treatment. In the case of scores ≥ 10, the individual was further tested with the Six-Minute Walk Test. In the case of a performance ≥ 350 Mt, the subject was prescribed a high-level program; otherwise they were assigned to a low-level treatment.

All included individuals were subjected to the Short Physical Performance Battery (SPPB) to also evaluate balance and fall risk; based on the result obtained, they subsequently carried out a Six-Minute Walk Test (6MWT) to evaluate their functional capacity. The subjects were thus categorized into two groups based on the tests results: low level or high level (Figure 2). Subjects unable to perform a 6MWT for clinical and/or functional reasons performed only the SPPB.

The program included daily 30 min moderate-intensity aerobic training using a pedal exerciser and twice-weekly 30 min strength training using adjustable dumbbells and ankle weights according to the low- or high-intensity categorization of the individual (Supplementary Table S2).

The patients undertook daily aerobic training sessions using a pedal exerciser, as well as strength training using ankle weights and dumbbells adjustable from 0.5 kg to 2.5 kg, twice a week on non-consecutive days for 30 min per session.

The loads were modulated by monitoring heart rate and the Borg scale, ensuring patients were receiving pharmacological therapy and verifying the absence of adverse symptoms. The treatment program was delivered via tele-rehabilitation using a dedicated platform.

For the high-level exercise group, the intensity was tailored to 70–80% of the theoretical maximum heart rate and with a Borg score of 3 to 5 on the CR10 scale, as this is more easily understood than the Borg RPE scale.

For the low-level exercise group, the targets were lower: 40–70% of the theoretical maximum heart rate, but with the same weekly frequency, starting with 10 min per session and increasing to 30 min if tolerated.

The intensity of the exercises was reassessed at every session, i.e., twice a week.

REMOTE PHYSIOTHERAPY TREATMENT PROTOCOL

1. Connect online with the patient via the platform;
2. Instruct the patient to wear the monitoring devices the patient has previously been trained to use;
3. Take the basal parameters with the patient sitting at rest for at least 5 min: detect heart rate, peripheral saturation, basal pressure and subjective sensation of effort using the Borg CR10 scale and enter them into a specific database;
4. Remote training with exercises based on FITT principles: frequency, intensity, time, type and progression of the exercise according to the patient’s functional level;
5. Take vital signs at the end of the session with the patient seated.

LOW LEVEL

Patients started training sessions with 30 min of callisthenic exercises, followed by a free effort reconditioning with the use of a seated pedal set without load for 10/15/20/30 min based on the patient’s clinical condition. In addition, a suggested free walk session of at least 10 and at most 30 min per day was set and monitored via a pedometer.

HIGH LEVEL

The sessions included 30 min of fatigue-tolerant resistance exercises with dumbbells and ankle cuffs with 2 sets of 15 repetitions. In addition, approximately 30 min of free endurance training on a cycle ergometer (EverFit Welly M), trying to achieve a Borg CR10 between 3/10 and 5/10, with 5 min of warm-up, 20 min of activity and 5 min of cool-down, was performed. The adequate workload was achieved gradually according to the FITT method (Supplementary Table S2).

### 2.20. Sample Size Estimation

From the literature, frailty at discharge is expected to be about 40% in the overall study population [17,18]. We expect frailty reversal in Groups A and B of 48% and 15%, respectively [8], so frailty at the end of follow-up is hypothesized to be 21% in Group A and 34% in Group B. To achieve 80% power in detecting a 13% difference in frailty prevalence between Groups A and B after the intervention, with an alpha error of 5%, a sample size of 370 subjects is needed. Considering about a 22% dropout rate, a total of 450 patients are required.

Considering that recent trials do not consider frailty as a binary outcome, but as a continuous variable [23], and that an improvement of 1 unit in the EFT scale is associated with a 28% lower mortality incidence and thus represents a clinically meaningful phenomenon [11], we also calculated the sample size needed to observe a difference in the mean level of EFT between the two groups. Considering that: 1—using real data collected in our units in other similar studies, the mean EFT at baseline is 2.25 ± 1.25; 2—assuming an improvement of 1 unit (minimal clinically important difference, i.e., the smallest difference in score that benefits the patient) and therefore a mean score at the end of the study of 1.25 ± 1.25 [11]; 3—assuming a power of 90% and alpha of 5%; and 4—assuming a 30% improvement in the control group treated with the standard of care (and thus expecting a mean EFT of 1.95 in this group) and maintaining the same SD for all, it follows that 136 individuals are needed to observe a difference in the mean level of EFT score at the end of the trial. Considering also a 22% possible dropout rate, a sample size of 166 people is needed for this analysis. The same calculation using the SPPB as the metric of interest provided similar results.

### 2.21. Randomization

Patients were randomly assigned in a 1:1 ratio to the intervention or control group through permuted-block randomization with varying block sizes. Randomization lists were center-specific and they were created using SAS^®^ Proc Plan permuted-block randomization. A list was created for each center. The lists were created with block sizes of 2, 4, and 6. The order of block permutations was then randomized within each list to avoid predictability. A seed was set for generating the list so that it could be reproduced if necessary. Patient allocation was performed via a centralized email system.

### 2.22. Data Collection and Statistical Analysis

Study data were collected in the eCRF of an electronic web-based database, where each patient is identified by a randomly assigned alphanumeric code that is center-specific.

Baseline data were analyzed through descriptive analyses. Results for the overall population and for both Groups A and B are presented. Categorical variables were presented as frequencies and percentages and compared between the two groups by Chi-square test of Fisher’s exact test. Continuous variables were summarized by mean ± standard deviation and tested for normality distribution by Kolmogorov–Smirnov test. Since all variables were not normally distributed, the non-parametric Wilcoxon–Mann–Whitney U test will be used to compare baseline variables between groups. The prevalence of frail patients in the two groups is indicated as an EFT score ≥ 3. For the planned analyses, mixed models, adjusted for unbalanced variables, will be used to account for different times to follow-up. Only people with available measures of EFT at both baseline and follow-up will be included in the analyses relative to the prevalence of frailty and EFT as a continuous variable, while only individuals with available measures of SPPB at both baseline and follow-up will be included in the analyses for this endpoint. For the other covariates, missing data will be imputed through regression models. Given the limited sample size, subgroup analyses are not planned.

All analyses were performed by using SAS Software version 9.4. Tests will be considered significant for a *p* value < 0.05.

### 2.23. Withdrawal from the Study (Or Modified Follow-Up)

A participant may withdraw from the study at any time at their own request, or may be withdrawn at any time at the discretion of the investigator for safety, behavioral, compliance, or administrative reasons.

### 2.24. Lost to Follow Up

A participant was considered lost to follow-up if they were repeatedly unable to be contacted by the study site.

### 2.25. Ethical Considerations

This study was conducted in accordance with consensus ethical principles derived from international guidelines including the Declaration of Helsinki and the Council for International Organizations of Medical Sciences’ International Ethical Guidelines, applicable ICH Good Clinical Practice Guidelines, and applicable laws and regulations.

The informed consent procedure included the use of simplified explanations of the digital tools involved, supported by visual aids and step-by-step demonstrations when necessary. Participants were encouraged to ask questions, and family caregivers could be involved in the discussion to facilitate comprehension and support decision-making. To verify participant understanding prior to obtaining consent, study staff provided additional explanations when needed and confirmed that participants clearly understood the study procedures, including the digital aspects, before consent was finalized. In addition, throughout the trial, participants had access to ongoing support from the research team to address potential difficulties with the digital components of the intervention. This support aimed to minimize barriers to participation and reduce the risk of digital exclusion.

The study and its amendments were approved by the Ethics Committee of IRCCS MultiMedica and CET Lombardia 5, Prot.nr.509/24 and Prot.nr.353/25. This protocol was deposited in Zenodo and is accessible at https://zenodo.org/records/18632447 (accessed on 13 February 2026).

## 3. Results

**Figure 3:**
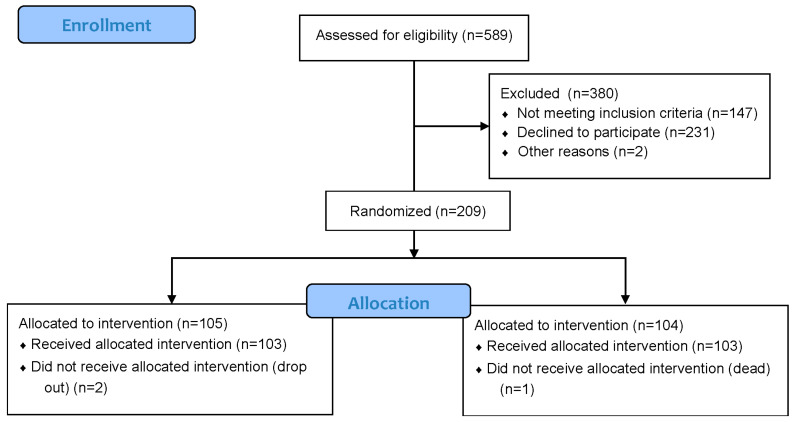
CONSORT flowchart of people screened, excluded and included in the trial.

From May 2024 to December 2025, 589 individuals were assessed for eligibility at the two centers. Among them, 147 patients were excluded because they met the exclusion criteria: more than one third (39%) were unable to use electronic devices and 21% were clinically unstable. Among the minor reasons for exclusion were MMSE < 15 (6%) and age < 65 years (4%). Therefore, we screened 442 individuals with recent ACS and AHF who fulfilled the inclusion criteria and were considered eligible for the trial. Among those eligible, 233 declined to participate in the study while two individuals could not be enrolled because they died during hospitalization. The most common reason for declining participation in the trial was the perceived complexity of being compliant with a demanding program, or the willingness to learn how to use the dedicated eHealth platform, a motivation cited by 46% of the eligible individuals approached. Overall, 19% complained about the lack of a caregiver. The remaining 209 people were enrolled in the study and assigned to one of the two groups through randomization. Two patients assigned to the control group dropped out before completing exams or questionnaires and one subject in the intervention group died before discharge (Figure 3). We compared included subjects with the subgroup of subjects who declined to participate with complete data about age, sex and type of cardiac event. The included population was representative of the eligible individuals in terms of age and sex but patients who declined were more likely to have a diagnosis of AHF compared with included individuals (58% vs. 42%, *p* = 0.0078).

Among people undergoing randomization, 103 were assigned to the eHealth intervention group and 103 to the standard-of-care control group. The baseline demographic and clinical characteristics of the two groups are presented in Table 1 while the overall characteristics of the population with the relative number of observations for each variable are shown in Table 2. Clinical characteristics were comparable in the two groups, with the exception of sex and SPPB (Table 1). Overall, the mean (±SD) age of the patients was 77 ± 9 years, and 145 subjects (70.4%) were male. The mean BMI was 25.8. More than half of the patients had ACS (55.3%). As expected, most of the people were receiving lipid-lowering medications (77.5%), antiplatelet drugs (56.4%), and one or more anti-hypertensive medications.

## 4. Discussion

The efficacy of eHealth-based, home-delivered CR interventions on frailty is debated [7]. To address this gap, we designed a randomized trial evaluating whether an eHealth CR program, compared with standard care, can reduce the prevalence or degree of frailty in patients >65 years hospitalized for AHF or ACS. Here we outline the trial protocol and report the baseline characteristics of the enrolled participants.

The number of enrolled individuals is lower than the planned sample size for the primary outcome, but sufficient for the co-primary outcomes. Thus, the study will be powered to detect a difference in the mean level of frailty but not to observe a difference in the prevalence of frailty considered as a binary variable, unless a higher-than-expected efficacy of the intervention emerges. Indeed, the study now reaches a power of 55% for the primary outcome when considering an expected difference of 13% in the final prevalence of frailty, while a difference of 17% in the prevalence of frailty would be needed to obtain a significant result. This aspect is of relevance, especially in case of non-significant findings.

The lower-than-expected enrollment capacity is attributable to the high rate of refusal to participate due to the reported inability or fear of using technological devices such as a tablet. These phenomena commonly, referred to as “digital illiteracy” and “technophobia”, are common in the oldest old and particularly pervasive in Italy [24]. As a result, we had to amend the protocol after the start of the study to lower the cutoff for age of inclusion in the study. We believe that this does not represent an issue since, as discussed, frailty represents a continuum with a broad spectrum of conditions that should be addressed as early as possible and not only when severe comorbidities are present [9,10]. In this respect, this consideration also emphasizes the relevance of the results, which will demonstrate whether an eHealth-based, multidomain intervention is able to affect the degree of frailty when considered as a continuous, and not binary, variable.

Independently of the results that we will observe, it is important to note that digital illiteracy represents a key limit for the wide diffusion and implementation of such programs in real-life scenarios. We observed that roughly 20% of the screened individuals could not be enrolled due to the observed inability to use simple technological devices, while almost half of those screened refused to participate due to a lack of willingness to engage in a home-based program or for a refusal to learn how to use the telemedicine software. Notably, the idea of using remote intervention based on telemedicine and telemonitoring was conceived to provide an alternative for older people with a high degree of disability and thus those who were struggling to participate in in-person programs and visits. Thus, even if the eHealth approach is effective, it will remain of limited usefulness if it cannot be implemented in a wide range of individuals. Even though common sense suggests that future generations of old populations, who grew up in the technological era, will not face such issues, older subjects living with these limitations at present should be helped to learn basic technological skills to have access to such programs.

Another aspect to be considered is that we opted to enroll people with either acute HF or ACS since they are both post-acute cardiac events. However, individuals with these conditions may differ in terms of baseline functional trajectory, symptom limitations (e.g., dyspnea/fatigue burden), medication profiles, contraindications, and expected recovery patterns. This aspect might generate heterogeneity in the response to the intervention.

Relative to the composition of the groups, these are well balanced, as expected by the randomization process. The only variables not balanced are sex and the SPPB. Thus, the analyses will be adjusted for such covariates, given their known relationship with the primary outcomes [25]. Cox models and ANCOVA, both adjusted for these unbalanced variables, were used to compare the prevalence and the degree of frailty, respectively. A sensitivity analysis using propensity score matching was also performed. In the active arm of the trial, 45 people performed low-level-intensity exercises and the others underwent a high-level program. Given this limited sample, subgroup analyses are not adequately powered and cannot explore whether the intervention is more effective in people with a low SPPB score, despite the prior literature suggesting that the frailest individuals (i.e., SPPB < 10) would show greater improvement [26].

## 5. Conclusions

We have described the full protocol of a trial testing whether a multidomain eHealth-based intervention is able to revert or limit frailty progression in people with CVD aged older than 65 years. Baseline data of the 206 included individuals are also reported.

## Figures and Tables

**Table 1:** Baseline characteristics of the included participants allocated to the two groups.

Variable	Intervention Group *n* = 103	Control Group *n* = 103	*p*-Value
Acute Coronary Syndrome	60 (58.25%)	54 (52.43%)	0.400
Acute Heart Failure	43 (41.75%)	49 (47.57%)	
EFT	1.69 ± 1.19	1.89 ± 1.23	0.298
Frailty prevalence (EFT ≥ 3)	30 (29.13%)	33 (32.04%)	0.650
SPPB	9.11 ± 3.46	8.27 ± 3.55	**0.046**
Male Sex	83 (80.58%)	62 (60.19%)	**0.001**
Age	76.96 ± 7.17	77.6 ± 10.38	0.220
BMI [kg/m^2^]	26.08 ± 4.44	25.45 ± 4.82	0.392
Systolic Blood Pressure [mmHg]	115.69 ± 13.62	116.67 ± 16.99	0.909
Diastolic Blood Pressure [mmHg]	68.47 ± 8.59	69.01 ± 9.16	0.829
HR [bpm]	71.13 ± 12.23	70.49 ± 10.93	0.962
Hemoglobin [g/dL]	12.21 ± 1.92	12.16 ± 1.63	0.973
Albumin [g/dL]	4.66 ± 5.29	3.85 ± 3.07	0.460
Total Cholesterol [mg/dL]	132.69 ± 43.1	132.57 ± 37.85	0.849
LDL-cholesterol [mg/dL]	72.69 ± 36.15	70.27 ± 31.5	0.927
HDL-cholesterol [mg/dL]	39.16 ± 11.63	43.29 ± 31.49	0.580
Triglycerides [mg/dL]	118.81 ± 79.14	110.57 ± 43.26	0.674
Creatinine [mg/dL]	1.27 ± 0.52	1.36 ± 0.62	0.255
eGFR [mL/min]	71.95 ± 21.22	67.91 ± 21.28	0.308
Beta-blocker	83 (83.84%)	81 (81.82%)	0.706
ACE*i*/ARB/ARNI	67 (68.37%)	61 (61.62%)	0.321
MRAs	70 (70%)	71 (71%)	0.877
Ivabradine	1 (1.05%)	2 (2.04%)	1.000 *
Aspirin	70 (70%)	56 (57.14%)	0.060
Other Antiplatelet Agents	58 (59.79%)	53 (53%)	0.336
Oral Anticoagulant Therapy	28 (29.17%)	26 (27.37%)	0.783
Diuretics	67 (66.34%)	59 (59%)	0.282
Metformin	13 (13.13%)	10 (10%)	0.490
Insulin	12 (12.24%)	13 (13.27%)	0.831
SGLT-2*i*	51 (51%)	50 (50.51%)	0.944
GLP-1 RA	17 (17.89%)	14 (14.58%)	0.535
Statins	80 (79.21%)	75 (75.76%)	0.559
Ezetimibe	65 (65%)	56 (56%)	0.193
PCSK9-*i*	1 (0.99%)	0 (0%)	1.000 *

Data are presented as mean ± SD for continuous variables and as number (percentage) for categorical variables. *p* values derive from Mann–Whitney U test for continuous variables and from Chi-square test for categorical variables. * Differences between the two groups were analyzed using Fisher’s exact test. Significant differences are highlighted in bold. EFT: Essential Frailty Toolset; SPPB: Short Physical Performance Battery; BMI: Body Mass Index; HR: heart rate; eGFR: estimated Glomerular Filtration Rate; ACE*i*: Angiotensin-Converting Enzyme inhibitors; ARB: Angiotensin Receptor Blockers; ARNI: Angiotensin Receptor–Neprilysin Inhibitors; MRAs: Mineralocorticoid Receptor Antagonists; SGLT2-inhibitor: Sodium–Glucose Cotransporter 2 inhibitors; GLP-1 RA: Glucagon-Like Peptide-1 Receptor Agonists; PCSK9-*i*: Proprotein Convertase Subtilisin/Kexin Type 9 inhibitors.

**Table 2:** Baseline characteristics of overall population, along with the number (*n*) of available data for each variable.

Variable	Overall	*n*
Intervention Group	103 (50%)	206
Control Group	103 (50%)	
Acute Coronary Syndrome	114 (55.34%)	206
Acute Heart Failure	92 (44.66%)	
Frailty Prevalence EFT ≥ 3	63 (30.58%)	206
SPPB	8.71 ± 3.51	171
Males	145 (70.39%)	206
Age	77.28 ± 8.91	206
BMI [kg/m^2^]	25.77 ± 4.63	203
Systolic Blood Pressure [mmHg]	116.2 ± 15.42	150
Diastolic Blood Pressure [mmHg]	68.75 ± 8.87	150
HR [bpm]	70.79 ± 11.52	148
Hemoglobin [g/dL]	12.19 ± 1.77	205
Albumin [g/dL]	4.26 ± 4.36	198
Total Cholesterol [mg/dL]	132.63 ± 40.44	189
LDL-cholesterol [mg/dL]	71.53 ± 33.92	171
HDL-cholesterol [mg/dL]	41.22 ± 23.71	185
Triglycerides [mg/dL]	114.73 ± 63.92	186
Creatinine [mg/dL]	1.31 ± 0.57	202
eGFR [mL/min]	70.26 ± 21.21	81
Beta-blocker	164 (82.83%)	198
ACE*i*/ARB/ARNI	128 (64.97%)	197
MRAs	141 (70.5%)	200
Ivabradine	3 (1.55%)	193
Aspirin	126 (63.64%)	198
Other Antiplatelet Agents	111 (56.35%)	197
Oral Anticoagulant Therapy	54 (28.27%)	191
Diuretics	126 (62.69%)	201
Metformin	23 (11.56%)	199
Insulin	25 (12.76%)	196
SGLT-2*i*	101 (50.75%)	199
GLP-1 RA	31 (16.23%)	191
Statins	155 (77.5%)	200
Ezetimibe	121 (60.5%)	200
PCSK9-*i*	1 (0.5%)	201

Data are presented as mean ± SD for continuous variables and as number (percentage) for categorical variables. EFT: Essential Frailty Toolset; SPPB: Short Physical Performance Battery; BMI: Body Mass Index; HR: heart rate; eGFR: estimated Glomerular Filtration Rate; ACE*i*: Angiotensin-Converting Enzyme inhibitors; ARB: Angiotensin Receptor Blockers; ARNI: Angiotensin Receptor–Neprilysin Inhibitors; MRAs: Mineralocorticoid Receptor Antagonists; SGLT2-inhibitor: Sodium–Glucose Cotransporter 2 inhibitors; GLP-1 RA: Glucagon-Like Peptide-1 Receptor Agonists; PCSK9-*i*: Proprotein Convertase Subtilisin/Kexin Type 9 inhibitors.

## Data Availability

The data presented in this study are available on request from the corresponding author. The data are not publicly available due to privacy and ethical restrictions.

## Supplementary Materials

The following supporting information can be downloaded at: https://www.mdpi.com/article/10.3390/jcm15072573/s1, Table S1: Schedule of activities; Table S2: Remote Physiotherapy Treatment.

## References

[1] Kulmala J., Nykänen I., Hartikainen S. (2014). Frailty as a predictor of all-cause mortality in older men and women. Geriatr. Gerontol. Int..

[2] Buurman B.M., Hoogerduijn J.G., de Haan R.J., Abu-Hanna A., Lagaay A.M., Verhaar H.J., Schuurmans M.J., Levi M., de Rooij S.E. (2011). Geriatric conditions in acutely hospitalized older patients: Prevalence and one-year survival and functional decline. PLoS ONE.

[3] Ijaz N., Buta B., Xue Q.L., Mohess D.T., Bushan A., Tran H., Batchelor W., deFilippi C.R., Walston J.D., Bandeen-Roche K. (2022). Interventions for Frailty Among Older Adults with Cardiovascular Disease: JACC State-of-the-Art Review. J. Am. Coll. Cardiol..

[4] Sirven N., Dumontet M., Rapp T. (2020). The dynamics of frailty and change in socio-economic conditions: Evidence for the 65+ in Europe. Eur. J. Public Health.

[5] Ambrosetti M., Abreu A., Corrà U., Davos C.H., Hansen D., Frederix I., Iliou M.C., Pedretti R.F.E., Schmid J.P., Vigorito C. (2021). Secondary prevention through comprehensive cardiovascular rehabilitation: From knowledge to implementation. 2020 Update. A position paper from the Secondary Prevention and Rehabilitation Section of the European Association of Preventive Cardiology. Eur. J. Prev. Cardiol..

[6] Scherrenberg M., Marinus N., Giallauria F., Falter M., Kemps H., Wilhelm M., Prescott E., Vigorito C., De Kluiver E., Cipriano G. (2023). The need for long-term personalized management of frail CVD patients by rehabilitation and telemonitoring: A framework. Trends Cardiovasc. Med..

[7] Esfandiari E., Miller W.C., Ashe M.C. (2021). The Effect of Telehealth Interventions on Function and Quality of Life for Older Adults with Pre-Frailty or Frailty: A Systematic Review and Meta-Analysis. J. Appl. Gerontol..

[8] Afilalo J., Lauck S., Kim D.H., Lefèvre T., Piazza N., Lachapelle K., Martucci G., Lamy A., Labinaz M., Peterson M.D. (2017). Frailty in Older Adults Undergoing Aortic Valve Replacement: The FRAILTY-AVR Study. J. Am. Coll. Cardiol..

[9] Howlett S.E., Rutenberg A.D., Rockwood K. (2021). The degree of frailty as a translational measure of health in aging. Nat. Aging.

[10] Newman A.B., Blackwell T.L., Mau T., Cawthon P.M., Coen P.M., Cummings S.R., Toledo F.G.S., Goodpaster B.H., Glynn N.W., Hepple R.T. (2024). Vigor to Frailty As a Continuum-A New Approach in the Study of Muscle, Mobility, and Aging Cohort. J. Gerontol. A Biol. Sci. Med. Sci..

[11] Solomon J., Moss E., Morin J.F., Langlois Y., Cecere R., de Varennes B., Lachapelle K., Piazza N., Martucci G., Bendayan M. (2021). The Essential Frailty Toolset in Older Adults Undergoing Coronary Artery Bypass Surgery. J. Am. Heart Assoc..

[12] Pavasini R., Guralnik J., Brown J.C., di Bari M., Cesari M., Landi F., Vaes B., Legrand D., Verghese J., Wang C. (2016). Short Physical Performance Battery and all-cause mortality: Systematic review and meta-analysis. BMC Med..

[13] Guralnik J.M., Ferrucci L., Simonsick E.M., Salive M.E., Wallace R.B. (1995). Lower-extremity function in persons over the age of 70 years as a predictor of subsequent disability. N. Engl. J. Med..

[14] Guralnik J.M., Simonsick E.M., Ferrucci L., Glynn R.J., Berkman L.F., Blazer D.G., Scherr P.A., Wallace R.B. (1994). A short physical performance battery assessing lower extremity function: Association with self-reported disability and prediction of mortality and nursing home admission. J. Gerontol..

[15] Short Physical Performance Battery (SPPB). https://www.nia.nih.gov/research/resource/short-physical-performance-battery-sppb.

[16] Holland A.E., Spruit M.A., Troosters T., Puhan M.A., Pepin V., Saey D., McCormack M.C., Carlin B.W., Sciurba F.C., Pitta F. (2014). An official European Respiratory Society/American Thoracic Society technical standard: Field walking tests in chronic respiratory disease. Eur. Respir. J..

[17] Mahoney F., Barthel D. (1965). Functional evaluation: The Barthel index. Md. State Med. J..

[18] Miller M.D., Paradis C.F., Houck P.R., Mazumdar S., Stack J.A., Rifai A.H., Mulsant B., Reynolds C.F. (1992). Rating chronic medical illness burden in geropsychiatric practice and research: Application of the Cumulative Illness Rating Scale. Psychiatry Res..

[19] Folstei Guigoz Y., Vallas B.J., Garry P.J. (1994). Mini Nutritional Assessment: A practical assessment tool for grading the nutritional state of older patients. Facts Res. Gerontol..

[20] Folstein S.E., McHugh P.R. (1975). “Mini Mental State” a practical method for grading the cognitive state of patients for the clinicians. J. Psychiatr. Res..

[21] Sheikh J.I., Yesavage J.A. (1986). Geriatric Depression Scale (GDS): Recent evidence and development of a shorter version. Clin. Gerontol. J. Aging Ment. Health.

[22] Morisky D.E., Green L.W., Levine D.M. (1986). Concurrent and predictive validity of a self-reported measure of medication adherence. Med. Care.

[23] Jang I.Y., Jung H.W., Lee H.Y., Park H., Lee E., Kim D.H. (2020). Evaluation of Clinically Meaningful Changes in Measures of Frailty. J. Gerontol. A Biol. Sci. Med. Sci..

[24] Cittadini e Competenze Digitali. https://www.istat.it/it/files/2023/06/cs-competenzedigitali.pdf.

[25] Holmberg M.J., Andersen L.W. (2022). Adjustment for Baseline Characteristics in Randomized Clinical Trials. JAMA.

[26] Baldasseroni S., Silverii M.V., Herbst A., Orso F., Di Bari M., Pratesi A., Burgisser C., Ungar A., Marchionni N., Fattirolli F. (2023). Predictors of physical frailty improvement in older patients enrolled in a multidisciplinary cardiac rehabilitation program. Heart Vessels.

