# Supplementary material for: Effectiveness on Frailty of an eHealth-Based Rehabilitation Program in Older People with Acute Heart Failure and/or Acute Coronary Syndrome: Study Protocol for a Randomized Trial and Baseline Data of Participants
Source: J Clin Med. 2026 Mar 27;15(7):2573. doi: 10.3390/jcm15072573 (PMC13073986; doi:10.3390/jcm15072573)
Supplement: Supplementary file 1 [file jcm-15-02573-s001.zip › jcm-4165279-supplementary.pdf]

Supplementary Materials for

Effectiveness on Frailty of an eHealth-Based Rehabilitation Program in Older People with Acute Heart Failure and/or Acute Coronary Syndrome: Study Protocol for a Randomized Trial and Baseline Data of Participants

Supplementary Table S1. Schedule of activities.

Activity	Step 1	Randomization<br>Step 2	Randomized treatment<br>Step 3				End of treatment<br>Step 4
Visit	1a	1b	1c			1d	2
Day	- 1<br>Day before discharge	0 Discharge Day					
Weeks			2	4	8	12	12-24
Signed informed consent form	x						
Inclusion/exclusion criteria	x						
Medical History	x						
Physical examinations/anthropometric meas.)	x		x			x	x
Collect blood sample		x	x			x	x
Questionaries'<br>ETF<br>EQ<br>ADL<br>GDS<br>MNA<br>CIRS<br>MMSE<br>Morisky	x		x			x	x
Six minute walk test	x		x			x	x
Events collections			x	x	x	x	x
treatment			x	x	x	x	

Schedule of planned activities for people screened and included in the trial.

Supplementary Table S2. Remote Physiotherapy Treatment.

	**LOW LEVEL**	**HIGH LEVEL**
**FREQUENCY**	2/WEEK	2/WEEK
**INTENSITY**	0 WATT	VARIABLE INTENSITY FROM 1 TO 8 (WATTS<br>TO BE CALCULATED) + RESISTANCE EXERCISES (0.5/2Kg DUMBBELLS + 0.5/2.5Kg ANKLETS)
**TIME**	10'/30'	30'/45'
**TYPE**	CHALISTENICS + PEDALS + FREE WALK	RESISTANCE (Sequence of 8 strengthening exercises with variable weights, 1 series of 15 repetitions):<br>1. AB/ADDUCTORS: from a standing position open your legs alternately<br>2. DELTOIDS: open/close arms with elbows extended<br>3. HIP FLEXORS: from a standing position, raise one knee and then the other to 90 degrees<br>4. TRICEPS: starting from the shoulders, rise to the ceiling until the elbows are straightened<br>5. QUADRICEPS: while sitting, alternately straighten one knee and then the other with your hammer feet; the thigh remains supported on the chair<br>6. BICEPS: start with elbows extended, bring the weights to the shoulders together<br>7. HAMLET BICEPS: from a standing position, alternately bend your legs at 90 degrees towards your buttocks<br>8. SHOULDER ROTATORS: elbows flexed at 90 degrees, the weights which when together open outwards keeping the elbows close to the chest<br>+PEDALS

Detail of the individualized, remote, physiotherapy treatment according to the ability of the individual in performing high or low frequency exercise.
